# A comparison of high-mobility group-box 1 protein, lipopolysaccharide-binding protein and procalcitonin in severe community-acquired infections and bacteraemia: a prospective study

**DOI:** 10.1186/cc5967

**Published:** 2007-07-11

**Authors:** Shahin Gaïni, Ole G Koldkjær, Holger J Møller, Court Pedersen, Svend S Pedersen

**Affiliations:** 1 Department of Infectious Diseases, Odense University Hospital, Søndre Boulevard 29, DK-5000 Odense C, Denmark; 2 Department of Clinical Biochemistry, Sønderborg Hospital, Sønderborg, Denmark; 3 Department of Clinical Biochemistry, AS-NBG Aarhus University Hospital, Aarhus, Denmark

## Abstract

**Introduction:**

High-mobility group box-1 protein (HMGB1) has been known as a chromosomal protein for many years. HMGB1 has recently been shown to be a proinflammatory cytokine with a role in the immunopathogenesis of sepsis. Lipopolysaccharide-binding protein (LBP) has a central role in the innate immune response when the host is challenged by bacterial pathogens. Procalcitonin (PCT) has been suggested as a marker of severe bacterial infections and sepsis. The aim of the present study was to investigate levels of HMGB1, LBP and PCT in a well-characterised sepsis cohort. The study plan included analysis of the levels of the inflammatory markers in relation to the severity of infection, to the prognosis and to the ability to identify patients with bacteraemia.

**Methods:**

Patients suspected of having severe infections and admitted to a department of internal medicine were included in a prospective manner. Demographic data, comorbidity, routine biochemistry, microbiological data, infection focus, severity score and mortality on day 28 were recorded. Plasma and serum were sampled within 24 hours after admission. Levels of all studied markers (HMGB1, LBP, PCT, IL-6, C-reactive protein, white blood cell count and neutrophils) were measured with commercially available laboratory techniques.

**Results:**

A total of 185 adult patients were included in the study; 154 patients fulfilled our definition of infection. Levels of HMGB1, LBP and PCT were higher in infected patients compared with a healthy control group (*P *< 0.0001). Levels of HMGB1, LBP and PCT were higher in the severe sepsis group compared with the sepsis group (*P *< 0.01). No differences were observed in levels of the inflammatory markers in fatal cases compared with survivors. Levels of all studied markers were higher in bacteraemic patients compared with nonbacteraemic patients (*P *< 0.05). PCT performed best in a receiver–operator curve analysis discriminating between bacteraemic and nonbacteraemic patients (*P *< 0.05). HMGB1 correlated to LBP, IL-6, C-reactive protein, white blood cell count and neutrophils (*P *< 0.001). LBP correlated to PCT, IL-6 and C-reactive protein (*P *< 0.001).

**Conclusion:**

Levels of HMGB1, PCT and LBP were higher in infected patients compared with those in healthy controls, and levels were higher in severe sepsis patients compared with those in sepsis patients. Levels of all studied inflammatory markers (HMGB1, LBP, PCT, IL-6) and infection markers (C-reactive protein, white blood cell count, neutrophils) were elevated among bacteraemic patients. PCT performed best as a diagnostic test marker for bacteraemia.

## Introduction

Sepsis is a serious clinical condition with a considerable morbidity and mortality [1]. Clinicians are in need of good diagnostic and prognostic markers to identify infected patients who could benefit from prompt empirical antibiotic therapy and other supportive therapy as early as possible. An increased knowledge of the immunopathogenesis of sepsis could have the potential of generating new diagnostic and treatment modalities for this serious condition.

High-mobility group-box 1 protein (HMGB1) is a nuclear chromosomal protein [2,3]. A new role for HMGB1 has been explored in recent years. HMGB1 has been suggested to have an important role as a 'late-onset' proinflammatory cytokine [4,5]. HMGB1 was rediscovered in this role when cultures of macrophages were exposed to endotoxin [4]. Animal models confirmed these observations, and there has been considerable attention on this protein especially in relation to sepsis and rheumatoid arthritis [4]. Lipopolysaccharide-binding protein (LBP) is an acute-phase protein with an important role in the innate immune system [6,7]. For the past 15 years attention has been pointed at the inflammatory marker procalcitonin (PCT) [8,9], which has been associated with severe bacterial infections among adults and children [9].

The present study purpose was to examine levels of HMGB1, LBP and PCT in patients with sepsis of different severity, in bacteraemic patients and in relation to the outcome of the patients. Another purpose was to examine the diagnostic test abilities of HMGB1, LBP and PCT to predict bacteraemia. Finally, correlations between the examined markers were explored.

## Methods

### Patients

Patients were included in a prospective manner in the period January 2003–June 2005. The setting was a large department of internal medicine at Odense University Hospital. The hospital serves a local population of approximately 185,000 inhabitants. Inclusion criteria for the study were suspicion of sepsis by the doctor in charge, initiation of empirical treatment with antibiotics and, finally, blood sampling should be possible within 24 hours after admission. Exclusion criteria were age <18 years, earlier participation in the study or prior hospitalisation within 7 days before admission. Plasma and serum were sampled from the included patients within 24 hours after admission. The samples were processed and frozen at -80°C within 1.5 hours. The patients received a standard of care according to departmental guidelines. The project protocol was approved by the Ethics Committee of Fyns and Vejle Counties. Informed consent was obtained from all patients or from their close relatives.

The patients' baseline characteristics, demographic data, biochemical parameters, systemic inflammatory response syndrome (SIRS) criteria and severity score were obtained at the time of inclusion. Severity was assessed with the Sepsis-related Organ Failure Assessment Score [10]. Comorbidity was assessed with the Charlson index [11].

Patients were classified at the time of inclusion according to the SIRS criteria [12]. Severe sepsis was defined as the presence of sepsis and one or several of the following indices of organ dysfunction: Glasgow coma scale ≤ 14, PaO_2 _≤ 9.75 kPa, oxygen saturation ≤ 92%, PaO_2_/FiO_2 _≤ 250, systolic blood pressure ≤ 90 mmHg, systolic blood pressure fall ≥ 40 mmHg from baseline, pH ≤ 7.3, lactate ≥ 2.5 mmol/l, creatinine ≥ 177 μmol/l, doubling of creatinine in patients with known kidney disease, oliguria ≤ 30 ml/hour for >3 hours or ≤ 0.7 l/24 hours, prothrombin time ≤ 0.6 s (reference 0.70–1.30 s), platelets ≤ 100 × 10^9^/l, bilirubin ≥ 43 μmol/l, and paralytic ileus. Septic shock was defined as hypotension persisting despite adequate fluid resuscitation for at least 1 hour. If a patient had any comorbidity that could more probably explain one or more of the criteria for organ dysfunction stated above, then the patient could not be categorised as having severe sepsis.

Infection was categorised according to the following definitions: culture/microscopy of a pathogen from a clinical focus; positive urine dip test in the presence of dysuria symptoms; chest X-ray-verified pneumonia; infection documented with another imaging technique; obvious clinical infection (that is, erysipelas, wound infection); and identification of a pathogen by serology or by PCR. The classification of the status of infection was made by only one physician, who was blinded to all biochemical results.

### Laboratory assays

HMGB1 was measured in serum with a commercially available ELISA (HMGB1 ELISA kit; Shino-Test Corporation, Tokyo, Japan). The measuring range was 0.6–93.8 ng/ml. The range could be broadened by dilution of high samples. The coefficient of variation was 5% for samples larger than 10 ng/ml and was 10% for samples between 2 and 5 ng/ml. Recovery of HMGB1 in this ELISA has been reported to be 92–111% [13]. The detection limit of HMGB1 was 0.6 ng/ml.

PCT was measured in plasma with a time-resolved amplified cryptate emission technology assay (Kryptor PCT^®^; BRAHMS Aktiengesellschaft, Hennigsdorf, Germany). The functional detection limit was 0.06 ng/ml. LBP and IL-6 were measured in plasma with a chemiluminiscent immunometric assay (Immulite-1000^®^; DPC, Los Angeles, CA, USA). The detection limit of LBP was 0.2 μg/ml and the detection limit of IL-6 was 2 pg/ml.

C-reactive protein (CRP) was measured with an immunoturbidimetric principle (Modular P^®^; Roche, Mannheim, Germany). White blood cells and neutrophils were counted on a Sysmex SE 9000^® ^(TOA Corporation, Kobe, Japan).

Levels of HMGB1, PCT, LBP and IL-6 were previously measured in a control group consisting of 32 healthy hospital workers [14].

### Statistical analyses

Data are presented as the median and interquartile range or as the mean ± standard deviation. Significance testing was carried out using the Kruskal–Wallis test and Wilcoxon's two-sample test. A two-tailed *P *value < 0.05 was considered statistically significant.

Receiver–operator characteristic (ROC) curves and the area under the curve (AUC) were determined for HMGB1, LBP and PCT. The AUC values are reported with the 95% confidence interval. The method described by DeLong and colleagues was used as the significance test for ROC and AUC comparison [15]. We compared diagnostic test performance by comparing the AUCs and by comparing the specificities when the sensitivity was approximately 80%. The Spearman rank correlation test was used to determine correlations. HMGB1 levels below 0.6 ng/ml were assigned a value of 0.6 ng/ml for calculations. IL-6 measurements below 2 pg/ml were assigned a value of 2 pg/ml for calculations. All statistical calculations were performed in the STATA 8^® ^statistical software package (STATA Corporation, College Station, TX, USA).

## Results

### Patient characteristics

One hundred and eighty-five adult patients were initiated on empirical antibiotic sepsis treatment and were included in our study. One hundred and fifty-four of the patients fulfilled our definitions for infection. Thirty-one patients were excluded from analyses (no infection present *n *= 9, uncertain diagnosis *n *= 22). Patients included in this study were elderly with a burden of comorbidity.

The patients were divided into the following groups for analyses: infections without SIRS (*n *= 20), sepsis (*n *= 56), severe sepsis (*n *= 67) and septic shock (*n *= 11). They were also divided according to the outcome (survivors *n *= 138, fatal cases *n *= 16). Finally the patients were divided according to the presence of bacteraemia (infections without bacteraemia *n *= 120, bacteraemia *n *= 34). Pneumonia and urinary tract infections were the most common infections.

The baseline characteristics/outcome and infectious characteristics are presented in Tables 1 and 2.

**Table 1:** Baseline characteristics and outcome of the patients

Variable	Infection without systemic inflammatory response syndrome (*n *= 20)	Sepsis (*n *= 56)	Severe sepsis (*n *= 67)	Septic shock (*n *= 11)
Male	7	31	37	2
Female	13	25	30	9
Age (years)	56.8 ± 22.9	56.9 ± 16.8	61.9 ± 17.5	67.3 ± 12.8
Hospitalisation (days)	5.9 ± 2.9	10.4 ± 9.2	14.3 ± 11.1	26.7 ± 22.9
Mortality on day 28	1 (5)	3 (5.4)	9 (13.4)	3 (27.3)
Sepsis-related Organ Failure Assessment score	1.4 ± 1.5	1.5 ± 0.9	3.4 ± 2.1	5.2 ± 2.7
Charlson index	0.7 ± 0.9	1.4 ± 1.9	1.3 ± 1.6	2.7 ± 1.5
Haemoglobin (mmol/l)	7.9 ± 0.9	8.2 ± 1.4	8.3 ± 1.4	7.7 ± 2.2
Platelet count (× 10^9^/l)	309.3 ± 152.3	299.6 ± 177.2	247.9 ± 142.8	270.6 ± 178.6
Bilirubin (μmol/l)	13.8 ± 15	11.2 ± 4.9	19.2 ± 14.9	15.1 ± 11.3
Prothrombin time (s)	0.8 ± 0.2	0.8 ± 0.2	0.7 ± 0.3	0.8 ± 0.2
Creatinine (μmol/l)	101.9 ± 47.9	98.7 ± 28.9	165.7 ± 118.8	239 ± 92.8

Data presented as the absolute number (%) or the mean ± standard deviation.

**Table 2:** Microbiological and infection characteristics of the patients

Variable	Infection without systemic inflammatory response syndrome (*n *= 20)	Sepsis (*n *= 56)	Severe sepsis (*n *= 67)	Septic shock (*n *= 11)
Bacteraemia				
Gram-positive bacteria	0	3	17	2
Gram-negative bacteria	1	2	5	3
>1 pathogen involved	0	0	1	0
Focus of infection				
Meningitis	1	2	9	0
Pneumonia	5	18	32	6
Endocarditis	0	1	4	0
Pyelonephritis	2	6	4	1
Cystitis	4	6	10	2
Cholecystitis/cholangitis	1	1	3	0
Gastroenteritis	0	1	0	0
Skin/soft tissue infection	6	9	2	1
Bone/joint infection	0	3	1	0
Other	1	9	2	1

Data presented as the absolute number.

### Levels of HMGB1, LBP and PCT related to the severity of infection

**Figure 1:**
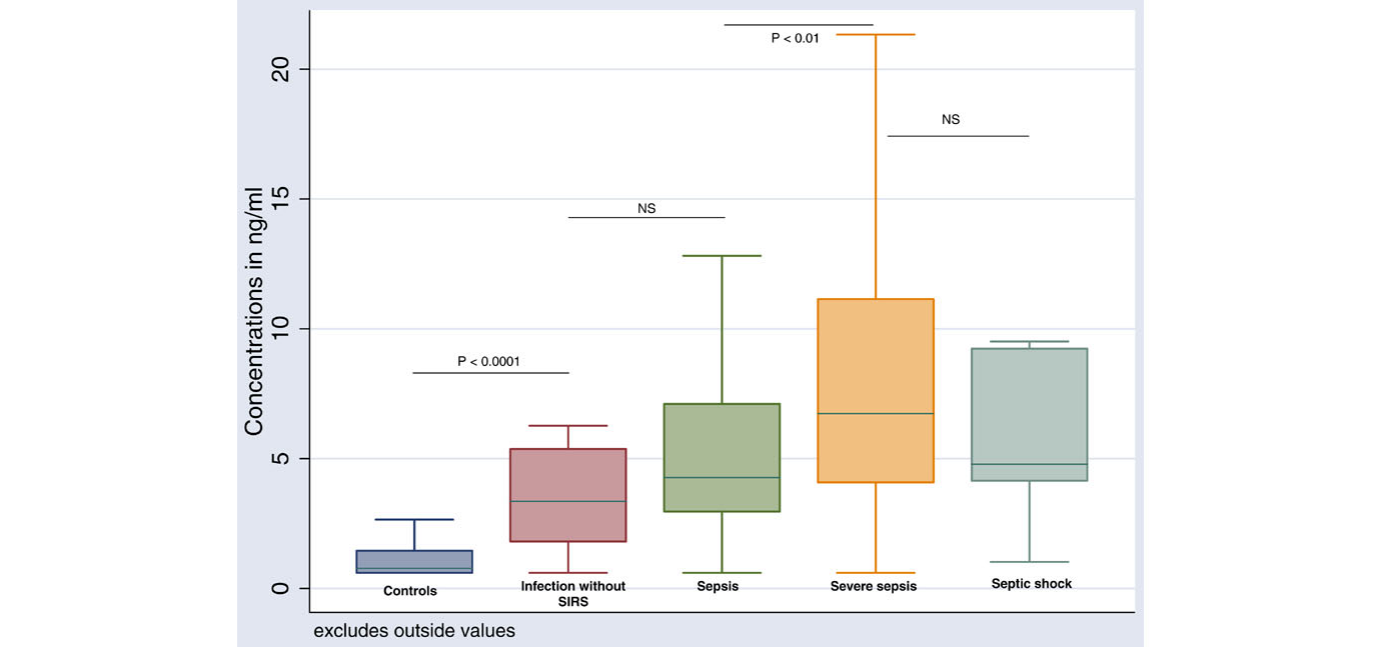
Boxplot of high-mobility group box-1 protein levels in healthy controls and infected patients. (Kruskal–Wallis, *P *< 0.001). NS, not significant.

HMGB1 levels were significantly higher among infected patients without SIRS compared with those in the healthy control group, and were significantly higher among severe sepsis patients compared with sepsis patients (*P *< 0.0001) (Figure 1 and Table 3). LBP levels were significantly higher among infected patients without SIRS compared with the healthy control group, were significantly higher among sepsis patients compared with infected patients without SIRS and, finally, were significantly higher among severe sepsis patients compared with sepsis patients (*P *< 0.05) (Table 3). PCT levels were significantly higher among infected patients without SIRS compared with the healthy control group, were significantly higher among severe sepsis patients compared with sepsis patients and, finally, were significantly higher among septic shock patients compared with severe sepsis patients (*P *< 0.05) (Table 3).

**Table 3:** Inflammatory markers related to the severity of infection

Variable	Healthy controls (*n *= 32)	Infection without SIRS (*n *= 20)	Sepsis (*n *= 56)	Severe sepsis (*n *= 67)	Septic shock (*n *= 11)	*P *value^a^
HMGB1 (ng/ml)						<0.001
Median	0.77	3.4	4.3	6.7	4.8	
IQR	0.6–1.5	1.8–5.4	2.9–7.1	4.1–11.1	4.1–9.2	
*P *value^b^		<0.0001	NS	< 0.01	NS	
Lipopolysaccharide-binding protein (μg/ml)						<0.001
Median	12.7	46.3	63.3	88.7	73.3	
IQR	9.8–16.8	23.9–64.7	44.8–87.9	61.3–129	62.3–91.8	
*P *value^b^		<0.0001	<0.05	<0.01	NS	
Procalcitonin (ng/ml)						<0.001
Median	0.05	0.15	0.4	4.4	46.1	
IQR	0.04–0.06	0.07–0.5	0.13–1.3	1.3–22.2	5.9–127.5	
*P *value^b^		<0.0001	NS	<0.0001	<0.05	
IL-6 (pg/ml)						<0.001
Median	3.4	23.6	46.9	120	6117	
IQR	3–3.7	12.3–46.1	13.9–102.9	35.9–661	110–10,212	
*P *value^b^		<0.0001	NS	<0.001	<0.01	
C-reactive protein (mg/l)						<0.01
Median		71	181	205	197	
IQR		28.5–199.5	120–255	126–306	146–270	
*P *value^b^			<0.01	NS	NS	
White blood cells (× 10^9^/l)						<0.05
Median		10.4	11.2	14.8	16.8	
IQR		7.2–13.9	8.5–16.8	10.5–18.5	7.4–25.3	
*P *value^b^			NS	NS	NS	
Neutrophils (× 10^9^/l)						<0.01
Median		7.8	8.9	12.4	15.5	
IQR		5.5–11.7	6.5–14.7	7.9–16.3	6.4–21.8	
*P *value^b^			NS	<0.05	NS	

Data presented as median and interquartile range (IQR). HMGB1, high-mobility group box-1 protein; SIRS, systemic inflammatory response syndrome. ^a^Kruskal–Wallis test. ^b^Compared with the previous group in the table (Wilcoxon's two-sample test); NS, not significant.

### Levels of HMGB1, LBP and PCT in survivors and in fatal cases

There were no statistically significantly differences in the levels of the examined inflammatory markers in surviving patients compared with those in fatal cases (Table 4). The IL-6 levels were marginally significantly higher among fatal cases (*P *= 0.06).

**Table 4:** Inflammatory markers in survivors and in fatal cases

Variable	Survivors (*n *= 138)	Fatal cases (*n *= 16)	*P *value^a^
High-mobility group-box 1 protein (ng/ml)	4.9 (2.9–9.1)	5.6 (3.4–14.2)	NS
Lipopolysaccharide-binding protein (μg/ml)	70.7 (45.6–112.3)	70.6 (57.1–89.7)	NS
Procalcitonin (ng/ml)	1.3 (0.17–8.9)	1.7 (0.4–12.2)	NS
IL-6 (pg/ml)	66.5 (21.2–174.5)	193.5 (47.9–589)	NS^b^
C-reactive protein (mg/l)	185 (109–263)	198 (130.5–274)	NS
White blood cells (× 10^9^/l)	13.2 (8.5–17.3)	14.7 (11.5–20.9)	NS
Neutrophils (× 10^9^/l)	11.2 (6.8–15.5)	12.7 (8.7–18.9)	NS

Data presented as median and interquartile range. NS, not significant. ^a^Wilcoxon's two sample test. ^b^*P *= 0.06.

### Levels of HMGB1, LBP and PCT in nonbacteraemic patients and in bacteraemic patients

The HMGB1, LBP and PCT levels were significantly higher among patients with bacteraemia compared with the nonbacteraemic patients (*P *< 0.05) (Table 5).

**Table 5:** Inflammatory markers in nonbacteraemic patients and in bacteraemic patients

Variable	Infections without bacteraemia (*n *= 120)	Bacteraemia (*n *= 34)	*P *value^a^
High-mobility group-box 1 protein (ng/ml)	4.6 (2.9–8.3)	7.3 (4.4–10.7)	<0.05
Lipopolysaccharide-binding protein (μg/ml)	65.3 (42.8–91.4)	101.4 (65.2–165.5)	<0.0001
Procalcitonin (ng/ml)	0.6 (0.15–3.9)	14.1 (2.9–31)	<0.0001
IL-6 (pg/ml)	50.3 (18.9–140)	211 (102–1833)	<0.0001
C-reactive protein (mg/l)	164 (90–245)	243 (172–306)	<0.001
White blood cells (× 10^9^/l)	12.7 (8.6–16.8)	15.8 (11–21.5)	<0.05
Neutrophils (× 10^9^/l)	10.5 (6.9–15.2)	13.6 (10.5–19.4)	<0.05

Data presented as median and interquartile range. ^a^Wilcoxon's two sample test.

### Diagnostic test abilities of HMGB1, LBP and PCT in diagnosing bacteraemia

**Figure 2:**
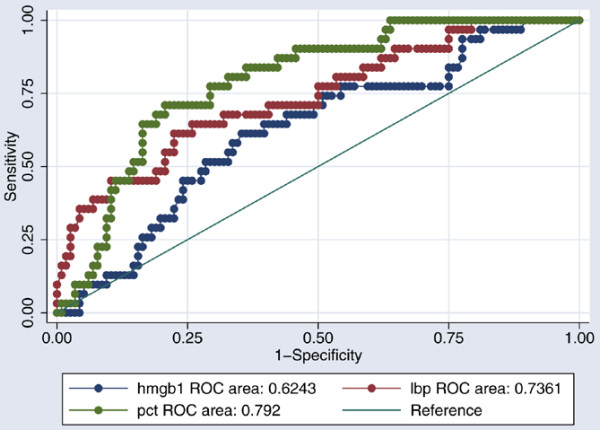
Receiver–operator characteristic curves comparing inflammatory markers. discriminating capabilities between nonbacteraemic patients and bacteraemic patients (*P *< 0.05).

PCT had a sensitivity of 80.7% and a specificity of 67.8% in diagnosing bacteraemia, with a cut-off level of 2.19 ng/ml (Table 6). In a ROC analysis examining the abilities to identify patients with bacteraemia, PCT performed best with an AUC of 0.79 (95% confidence interval: 0.73–0.88) (Figure 2). HMGB1 performed with an AUC of 0.62 (95% confidence interval: 0.51–0.73) in the analysis, and LBP presented an AUC of 0.74 (95% confidence interval: 0.65–0.85) (Figure 2).

**Table 6:** Specificity of the studied markers with cut-off levels corresponding to a sensitivity of approximately 80% in diagnosing bacteraemia

Variable	Cut-off level	Sensitivity (%)	Specificity (%)
High-mobility group-box 1 protein	4.2 ng/ml	79.4	45.0
Lipopolysaccharide-binding protein	64.6 μg/ml	79.4	50.0
Procalcitonin	2.19 ng/ml	80.7	67.8
IL-6	94.6 pg/ml	79.4	67.5
C-reactive protein	169 mg/l	79.4	51.3

### Correlations between the examined markers

HMGB1 correlated weakly to IL-6 and CRP, and correlated moderately to LBP, white blood cells and neutrophils (Table 7). LBP correlated weakly to IL-6, and correlated moderately to PCT and CRP (Table 7).

**Table 7:** Correlations between high-mobility group-box 1 protein (HMGB1)/lipopolysaccharide-binding protein (LBP) and the examined inflammatory markers

HMGB1 versus marker	Spearman's *r*	*P *value	LBP versus marker	Spearman's *r*	*P *value
LBP	0.3	<0.001	HMGB1	0.3	<0.001
Procalcitonin	0.15	NS	Procalcitonin	0.45	<0.0001
IL-6	0.18	<0.05	IL-6	0.29	<0.001
C-reactive protein	0.27	<0.001	C-reactive protein	0.64	<0.0001
White blood cells	0.39	<0.0001	White blood cells	0.11	NS
Neutrophils	0.39	<0.0001	Neutrophils	0.11	NS

NS, not significant.

## Discussion

HMGB1 has been known for many years as a chromosomal protein. In recent years there has been interest in HMGB1's role as a proinflammatory cytokine [4,5]. Animal models have shown that HMGB1 has an important role in immunopathogenesis in sepsis [4]. Administration of exogenous HMGB1 to septic animals increased mortality, and administration of antibodies against HMGB1 ameliorated the clinical outcome of septic animals [4]. HMGB1 has been characterised as a 'late-onset' proinflammatory cytokine involved in the late phases of the septic process, after the early induction of 'early-onset' proinflammatory cytokines such as TNFα and IL-1 [4,5]. Disappointing results in trials trying to suppress early proinflammatory pathways in sepsis have made HMGB1 an interesting target molecule in sepsis [4,5,16].

HMGB1 levels have been measured in several clinical sepsis cohorts [4,14,17-20]. Three of these studies used blotting methods [4,17,20] and three of the studies used ELISA techniques [14,18,19]. In the study by Wang and colleagues, patients with fatal sepsis had median HMGB1 levels of 84 ng/ml and surviving sepsis patients had median HMGB1 levels of 25 ng/ml [4]. In the study by Sunden-Cullberg and colleagues, the HMGB1 levels in critically ill patients remained elevated for up to 1 week, with mean levels of HMGB1 over 340 ng/ml after a 144-hour observation period [17]. In a study of community-acquired pneumonia by Angus and colleagues, median HMGB1 levels of 190 ng/ml were observed [20]. Much lower levels were seen in the three studies using HMGB1 ELISA techniques [14,18,19]. In the study by Hatada and colleagues, infected patients had median HMGB1 levels of 4.54 ng/ml [18]; Yasuda and colleagues, studying infected patients with severe acute pancreatitis, observed mean HMGB1 levels of 7.8 ng/ml [19]; and, finally, in a study performed by our group, the median HMGB1 level in mild sepsis was 2.14 ng/ml [14].

In the present study the HMGB1 levels were comparable with the latter three aforementioned studies using ELISA for HMGB1 measurements [14,18,19]. HMGB1 levels in the present study were higher in bacteraemic patients compared with those in nonbacteraemic patients and HMGB1 correlated to several proinflammatory markers (LBP, CRP, white blood cells and neutrophils). These correlations seem to confirm a proinflammatory role for HMGB1 in human sepsis. HMGB1 did not perform well in a ROC analysis examining its ability to identify bacteraemic patients, with an AUC of only 0.62. As mentioned earlier, levels of HMGB1 were much lower than levels reported in studies using blotting techniques. The reason for this is not clear. One possibility is that our patients who were recruited from an ordinary department of internal medicine were less ill compared with studies conducted on intensive care units. Another possibility is that we sampled patients in the early phase of disease (within 24 hours after admission), which perhaps could explain the low levels of a 'late-onset' proinflammatory cytokine such as HMGB1. Finally, the chosen laboratory technique might explain the low levels. The presence of interfering inhibitory factors/autoantibodies to HMGB1 in human serum could affect results of HMGB1 measurements with ELISA techniques [21]. It is still unknown whether the currently used assays detect biologically active HMGB1. This is an important issue for future studies focusing on HMGB1 levels and disease activity.

LBP is a protein with a central role in the innate immune response in both Gram-negative and Gram-positive infection when the host is challenged by an invading pathogen [6,7]. In Gram-negative infection, LBP carries the endotoxin lipopolysaccharide to the CD14 receptors on the monocyte-macrophage cell lineage [22,23]. CD14 receptors then interact with the Toll-like receptor 4, initiating cytokine production [22,23]. The lipotheichoic acid from pneumococci and staphylococci activates a cellular response through Toll-like receptor 2 [24]. This response can be enhanced by LBP and CD14 [7].

Several clinical studies have examined the levels of LBP in infected patients [25-29], in which the median levels of LBP were between 21.1 μg/ml and 59.7 μg/ml. Only one previous study has examined LBP's diagnostic test abilities in diagnosing Gram-negative bacteraemia [29]. The authors found a sensitivity of 100% and a specificity of 92% with a high cut-off level (46.3 μg/ml) for LBP. The study only included four patients with Gram-negative bacteraemia [29]. In the present study, the median levels of LBP were high compared with the previous studies. LBP levels in the present study were higher in bacteraemic patients compared with nonbacteraemic patients, and LBP correlated to several proinflammatory markers (HMGB1, PCT and CRP). LBP correlated to the severity of infection. LBP did not perform well in a ROC analysis examining its ability to identify bacteraemic patients, with an AUC of 0.74.

PCT is a protein involved in the immunopathogenesis of sepsis. Many different parenchymal cells are able to produce PCT when the host is challenged by a pathogen [30]. Animal models have shown that administration of exogenous PCT to septic animals increased mortality and administration of antibodies against PCT to septic animals protected against fatal outcome [31,32]. Elevated levels of PCT have been associated with several conditions, such as toxic shock syndrome, bacterial sepsis, postoperative infectious complications, meningitis, cholangitis, pancreatitis with infection, malaria and fungemia [33]. PCT has been shown to be a marker associated with the severity of sepsis [34-38]. Several previous studies have examined PCT's diagnostic test abilities in diagnosing bacteraemia [39-44]. These studies found AUCs between 0.71 and 0.85 [39-44]. In the present study the PCT levels increased with increasing severity of infection, with the highest levels in severe sepsis (median 4.4 ng/ml) and in septic shock (median 46.1 ng/ml). These data confirm findings from earlier studies showing that PCT is a severity marker in sepsis.

Our study data showed more than 20-fold higher PCT levels in bacteraemic patients compared with nonbacteraemic patients. The AUC of PCT in diagnosing bacteraemia was 0.79. This result regarding PCT's diagnostic test abilities in diagnosing bacteraemia confirms the abovementioned findings in previous studies. Diagnosis of bacteraemia at the present time is a relatively slow process, requiring up to several days of culturing/processing in the laboratory of microbiology. It is possible that a good biochemical marker for the presence of bacteraemia could have a role in stratifying patients to faster microbiological diagnosis with molecular diagnostic techniques. A broad-range PCR could perhaps be a possible strategy speeding up the species diagnosis in bacteraemia. PCT may have a role in identifying patients that could benefit from fast molecular diagnostics.

The strengths of the present study are its prospective design, the relatively large sample size, well-characterised patients and fast blood sampling after admission to the hospital. The study focused on patients admitted to a general department of internal medicine. Many previous studies examining immunological, prognostic and diagnostic markers in sepsis have focused upon critically ill patients on intensive care units. Most patients with infections and sepsis are, however, at the milder end of the sepsis spectrum and will be treated on general departments of internal medicine or surgery. The risk of work-up bias was reduced by classifying the infectious status of the patients without access to the biochemical laboratory results. The laboratory technicians were blinded from the clinical data. The risk of spectrum bias was reduced by using relatively broad inclusion criteria, including all age groups over 18 years, all kinds of infectious foci, different aetiology, different severity and comorbidity.

A drawback of the study design was the risk of an imperfect gold standard bias. Before inclusion of patients was begun, the criteria for infection and sepsis severity were established by the study group. These criteria were followed rigorously to minimise the risk of an imperfect gold standard bias. Patients with uncertain diagnosis were excluded for the same reason. Drawbacks of the present study, as in many other clinical sepsis studies, were the heterogeneity among included patients, a heavy burden of comorbidity, variable severity of disease, variation in infectious focus, variation in microbiological aetiology and different lengths of disease prior to hospitalisation.

## Conclusion

This is the largest prospective study that has been conducted regarding HMGB1 measurements in infections and sepsis. Elevated levels of HMGB1, LBP and PCT were associated with the presence of infection and with the presence of bacteraemia in patients with community-acquired infections. None of the examined inflammatory markers had prognostic abilities in identifying patients with fatal outcome. PCT had better diagnostic test abilities in diagnosing the presence of bacteraemia compared with HMGB1 and LBP. PCT could have a future role in identifying patients who would benefit from new faster molecular diagnostic techniques for diagnosing bacteraemia.

## Key messages

• HMGB1 is a proinflammatory cytokine in severe infections and bacteraemia.

• LBP and PCT are severity markers in severe infections and bacteraemia.

• PCT is a better diagnostic test marker for bacteraemia compared with HMGB1 and LBP.

## Abbreviations

AUC = area under the curve; CRP = C-reactive protein; ELISA = enzyme-linked immunosorbent assay; FiO_2 _= fraction of inspired oxygen; HMGB1 = high-mobility group box-1 protein; IL = interleukin; LBP = lipopolysaccharide-binding protein; PaO_2 _= partial pressure of arterial oxygen; PCR = polymerase chain reaction; PCT = procalcitonin; ROC = receiver–operator characteristic; SIRS = systemic inflammatory response syndrome; TNF = tumour necrosis factor.

## Competing interests

The authors declare that they have no competing interests.

## Authors' contributions

SG planned the study, wrote the protocol, collected and analysed the data, and wrote the report. OGK was responsible for the PCT, LBP and IL-6 analyses. HJM was responsible for the HMGB1 analyses. CP and SSP were involved in planning the study, in revising the manuscript and in practical clinical aspects. All authors read and approved the final manuscript.
